# Accessory breast granulomatous mastitis: A case report and mini-review of the literature

**DOI:** 10.3892/mi.2025.277

**Published:** 2025-10-23

**Authors:** Abdulwahid M. Salih, Ari M. Abdullah, Lana R.A. Pshtiwan, Sakar O. Arif, Zuhair D. Hammood, Shaban L. Tofiq, Hiwa O. Abdullah, Masty K. Ahmed, Harzal Hiwa Fatih, Meer M. Abdulkarim, Fahmi H. Kakamad

**Affiliations:** 1 Department of Scientific Affairs, Smart Health Tower, Sulaymaniyah 46001, Iraq; 2 Department of Pathology, Sulaymaniyah Teaching Hospital, Sulaymaniyah 46001, Iraq; 3 Department of Surgery, Tikrit Teaching Hospital, Tikrit, Saladin Governorate 34001, Iraq; 4 College of Medicine, University of Sulaimani, Sulaymaniyah 46001, Iraq; 5 Kscien Organization for Scientific Research (Middle East Office), Sulaymaniyah 46001, Iraq

**Keywords:** granulomatous mastitis, accessory breast, corticosteroids, breast inflammation

## Abstract

Granulomatous mastitis (GM) is a rare inflammatory condition that primarily affects the breasts, and its occurrence in accessory breast tissue is even rarer. The present report describes the case of a patient with GM in the accessory breast. A 43-year-old female patient presented with a 1-month history of pain in the left axilla and breast. An analysis of her medical and surgical history did not reveal any notable findings, but she had a history of four full-term pregnancies and a cumulative lactation period of 4 years. Upon a clinical examination, a palpable, ill-defined, tender mass was noted along with an accessory breast and nipple in the axilla. The diagnosis was chronic mastitis, and she was treated with oral corticosteroids, amoxicillin, cabergoline and analgesics. Her condition initially improved; however, the symptoms recurred 1 year later. A wide local excision of the left axillary tail was performed. The patient experienced marked improvement post-surgery and remained stable, with no recurrence, at the 1-year follow-up. In addition, in the present study, seven recent cases of GM were included for a brief literature review, involving patients aged 23 to 42 years. Of these cases, 6 cases did not have any notable medical histories. A total of 6 patients had a history of pregnancy, with an average lactation duration of 22.7 months. The right breast was affected in 6 cases. Pain and swelling were the most frequent symptoms. A conservative approach, which included antibiotics, corticosteroids and wound dressing was used for 5 patients. In total, 6 patients achieved recovery. On the whole, the present study demonstrates that accessory breast tissue can develop GM. While corticosteroids may provide favorable short-term results, they do not necessarily prevent recurrence, whereas surgical management may provide more durable long-term outcomes.

## Introduction

Granulomatous mastitis (GM) is a rare inflammatory condition of the breast that primarily affects women of childbearing age, often with a history of breastfeeding. GM is categorized into idiopathic or primary GM and secondary GM (1,2). It primarily involves the mammary gland, although in rare cases, it can develop in accessory breast tissue (2). While the exact cause of GM remains unclear, the leading hypothesis suggests an autoimmune origin. Various factors, including medications, diabetes, trauma and smoking, may trigger the inflammatory response. However, the strongest associations with GM are pregnancy, lactation and hyperprolactinemia. The disease accounts for <1% of breast biopsies (3). Despite being a benign disease, GM is frequently difficult to detect, as it often masquerades as breast carcinoma, which is the primary concern at the clinical stage (4). Its locally aggressive character causes long-term discomfort and distress for affected patients. Its non-specific imaging findings can lead to delayed diagnosis, misinterpretation and potentially unnecessary invasive procedures. Only a limited number of cases of GM in axillary breast tissue have been documented (1,2). The present report describes the case of a 43-year-old female patient with GM in the accessory breast. The report has been organized following the CaReL guidelines, and only reliable, peer-reviewed sources were included, while excluding any untrustworthy references or data (5,6).

## Case report

### Patient information

A 43-year-old lactating woman presented to the Breast Clinic at Smart Health Tower (Sulaymaniyah, Iraq) with a 1-month history of pain in the left axilla and breast. An analysis of her past medical and surgical history did not reveal any notable findings. She had a history of four full-term pregnancies and a cumulative lactation period of 4 years.

### Clinical examination

**Figure 1:**
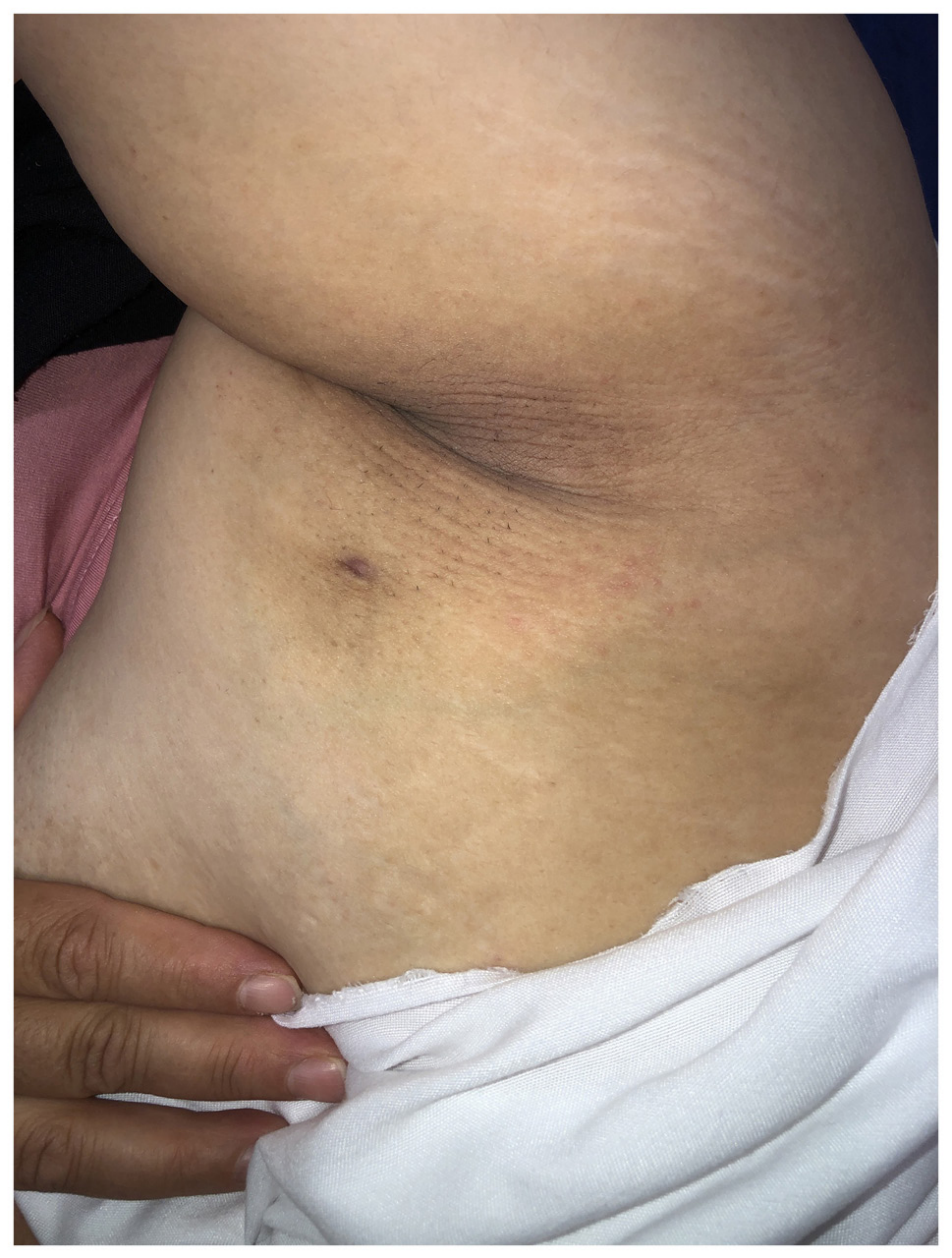
Image of the axillary region illustrating an area of hyperpigmentation with an indurated appearance. A small, isolated sinus tract is visible, indicating underlying chronic inflammation. The affected region corresponds to the axillary tail of Spence, where ectopic breast tissue is present. The surrounding skin appears slightly thickened, with mild erythema suggesting an ongoing inflammatory process.

The clinical examination revealed a palpable, ill-defined area of hardness with a firm consistency and tenderness upon palpation. Additionally, an axillary breast with a nipple was noted (Fig. 1).

### Diagnostic approach

**Figure 2:**
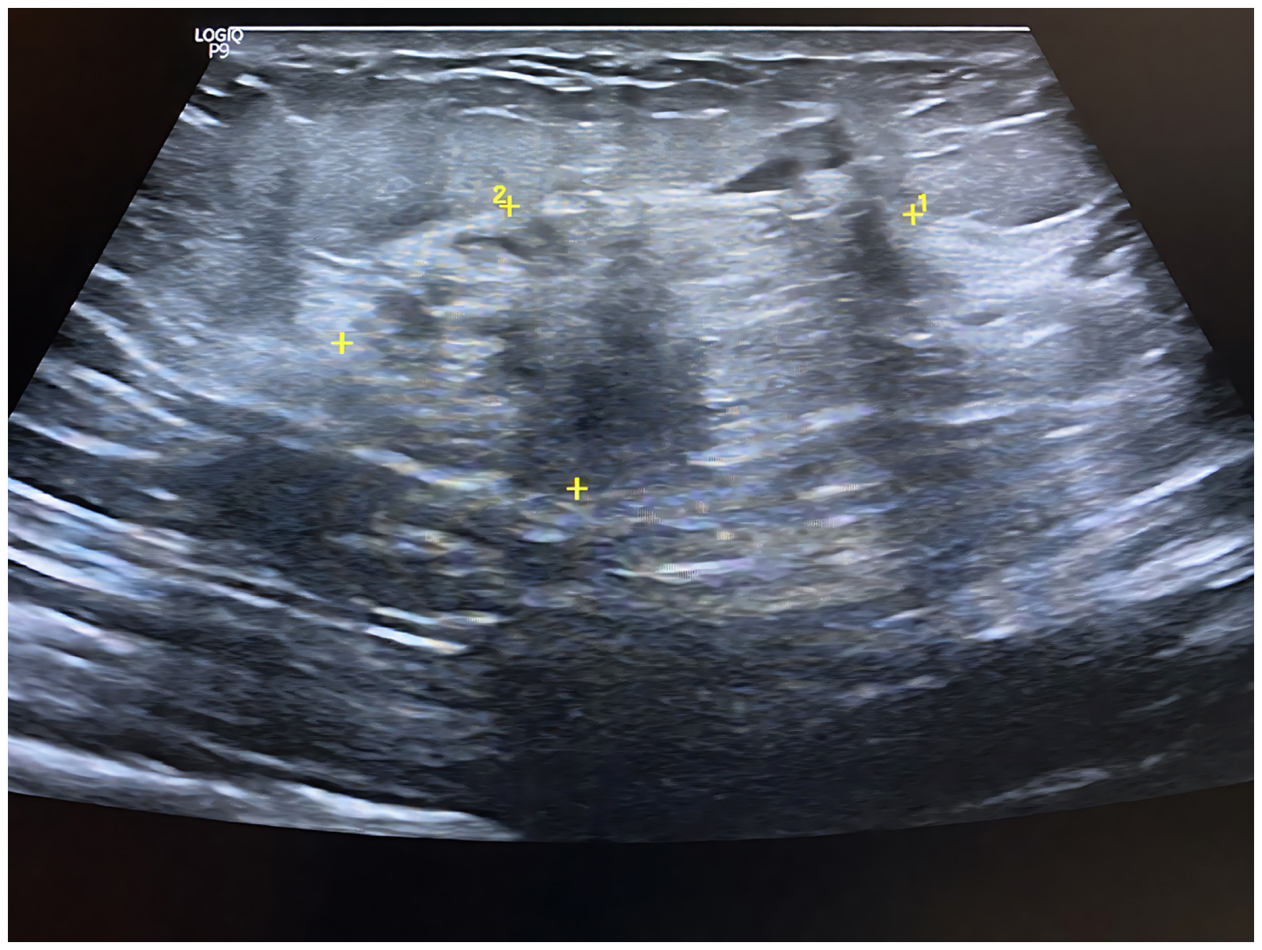
Grayscale ultrasound of the right axilla demonstrating accessory breast tissue with heterogeneous parenchymal echotexture and mild edema, consistent with mastitis. The numbers ‘1’ and ‘2’ designate the two orthogonal caliper sets used to measure the long- and short-axis of the lesion, respectively; the yellow crosses indicate the caliper endpoints. The recorded dimensions are 36x9 mm (long x short)

A breast ultrasonography demonstrated bilateral axillary breast tissue, more prominent on the left side, containing two distinct heterogeneous collections, the largest measuring 36x9 mm. Mild edema, skin sinuses and non-specific axillary nodes were also observed. These findings are consistent with chronic mastitis involving axillary breast tissue (Fig. 2) (1).

### Therapeutic intervention

**Figure 3:**
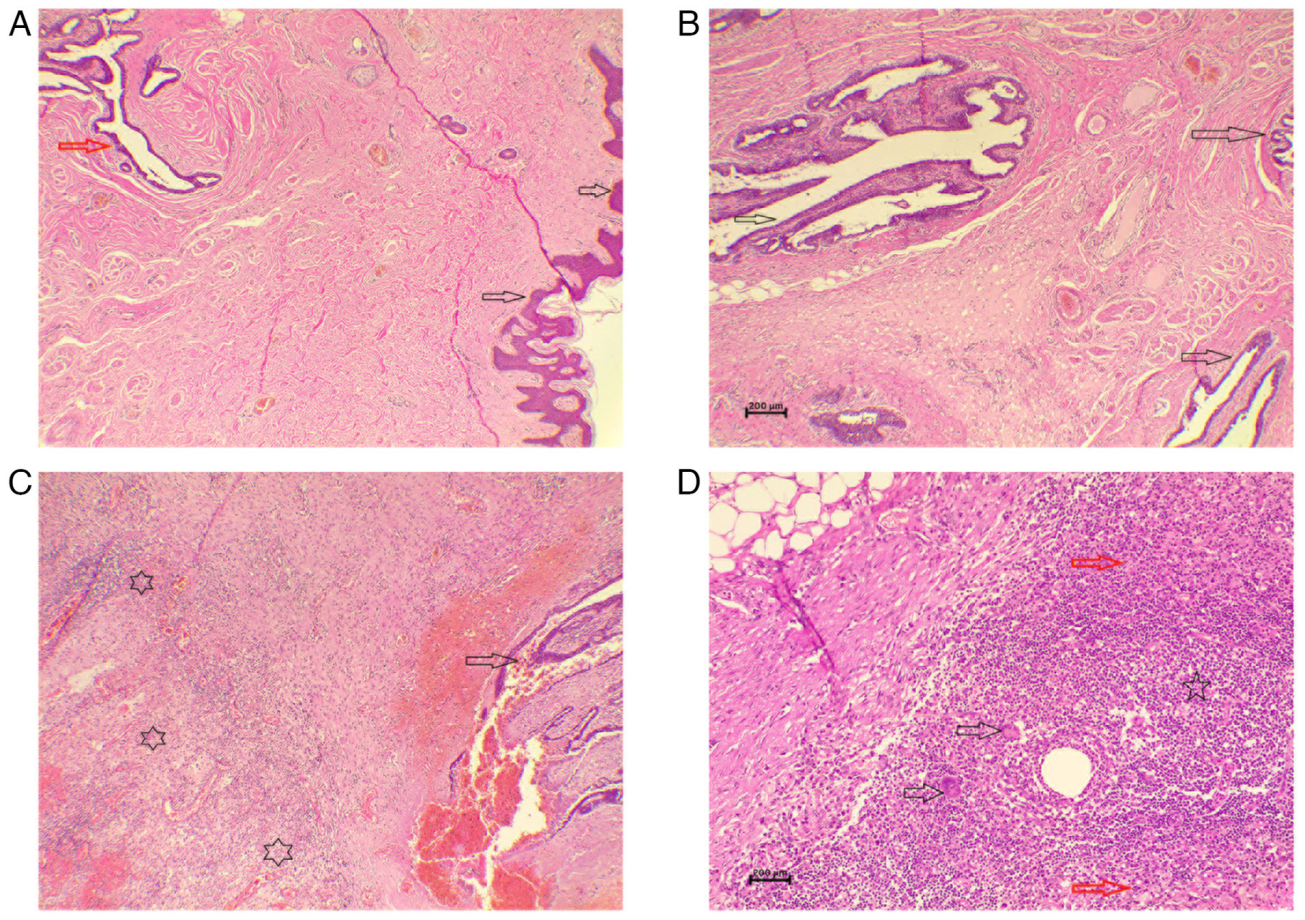
(A) Section of skin covered by benign epidermis (black arrows) with underlying tissue containing a lactiferous duct (red arrow). Hematoxylin and eosin staining; magnification, x4. (B) Higher magnification demonstrating multiple benign lactiferous ducts lined by ductal epithelial cells (black arrows). Hematoxylin and eosin staining; magnification, x10. (C) Section illustrating a benign lactiferous duct (black arrow) surrounded by tissue heavily infiltrated with mixed inflammatory cells and foamy macrophages (black stars). Hematoxylin and eosin staining; magnification, x4. (D) Higher magnification image illustrating multinucleated giant cells (black arrows) with ill-formed epithelioid histiocyte aggregates (red arrows) and dense mixed inflammatory cell infiltration (black star). Hematoxylin and eosin staining; magnification, x10.

The patient was initially diagnosed with periductal mastitis and managed medically with a tapering course of oral corticosteroids (prednisolone 10 mg once daily for 20 days, followed by 5 mg once daily for an additional 20 days; this was used to suppress the immune-mediated inflammatory process underlying GM and to reduce swelling, pain and disease activity), in combination with amoxicillin (1 g three times daily for 7 days, to cover potential secondary bacterial infection, which may complicate the course of GM, particularly when abscesses, fistulae, or skin involvement are present), cabergoline (0.5 mg once daily for 2 days, to suppress prolactin secretion) and analgesics (co-codamol 500 mg, two tablets as needed for pain control). She demonstrated a good clinical response to this regimen; however, the condition recurred 1 year later. Following multidisciplinary team discussion, a wide local excision of the left axillary tail was performed. A histopathological analysis of the excised specimen was performed on 5-µm-thick sections fixed in 10% neutral-buffered formalin for 24 h, embedded in paraffin, and stained with hematoxylin and eosin (Bio Optica Co.) for 1-2 min at room temperature. Examination under a light microscope (Leica Microsystems GmbH) revealed xanthogranulomatous inflammation involving the axillary accessory breast tissue, along with benign lymph nodes showing acute lymphadenitis (Fig. 3).

### Follow-up and outcome

At the 2-month follow-up time point, the symptoms of the patient had markedly improved, with no signs of recurrence. At the most recent annual follow-up following surgical excision, she remained in good health and recurrence-free.

## Discussion

Accessory breast tissue arises along the embryonic mammary ridge, extending from the axilla to the pubic region, and is susceptible to the same pathological conditions as normally located breast tissue (1). The frequently reported conditions in accessory breast tissue include cancer, mastitis, fibroadenomas, phyllodes tumors and fibrocystic changes (7). The leading hypothesis regarding the pathogenesis of GM suggests that an autoimmune response is initiated within the lobules of the breast parenchyma following ductal injury. This triggers a localized inflammatory reaction in the connective tissue, promoting the recruitment of macrophages and lymphocytes, ultimately resulting in a noncaseating granulomatous response (3).

The influence of ethnicity on GM remains a subject of debate. Vall *et al* (3) reported no specific ethnic predisposition. By contrast, the studies by Yuan *et al* (8) and Deng *et al* (9) indicated a higher prevalence of GM in Middle Eastern populations compared to Western countries. Consistent with the present case, GM predominantly affects women of reproductive age, particularly those with a history of breastfeeding. Among the 7 cases reviewed herein, 4 patients had a prior history of breastfeeding, with an average duration of 22.7 months (Table I) (1-4,7,10,11). However, Nakamura *et al* (7) and Rajendran *et al* (10) documented cases of GM in women without a history of breastfeeding.

Bilateral involvement in GM is rare, as the condition typically presents unilaterally (1). In the patient in the present study, a breast ultrasonography identified two distinct heterogeneous collections within the left axillary breast, accompanied by mild edema and skin sinuses. These imaging findings were consistent with the characteristic manifestations of GM, which commonly include mass formation, skin changes and the development of a sinus tract. Systemic symptoms, such as fever, remain uncommon in GM cases (1). Among the cases reviewed herein, all were unilateral, with fever reported in only 1 patient. Pain was the reported symptom in 6 cases, whereas erythema was observed in only 2 cases.

The diagnosis of GM requires a well-coordinated multidisciplinary approach involving clinicians, radiologists and pathologists, as demonstrated in the present case report. This is particularly critical given that cases with clinical deviance or coexistence with breast cancer have been reported, increasing the risk of misdiagnosis. Multidisciplinary collaboration not only helps to avoid such errors, but also facilitates earlier preoperative diagnosis and ensures the appropriate use of corticosteroid therapy in selected patients (8,12). Due to the absence of specific clinical or radiological characteristics, imaging findings may be non-specific. Mammography often reveals asymmetric density, while an ultrasound may detect irregular, heterogeneous masses suggestive of abscesses. However, these features are not unique to GM and can mimic other breast pathologies (1).

Although no standardized treatment exists for GM to date, at least to the best of our knowledge, steroids are frequently used to reduce lesion size. However, their use is associated with adverse effects, such as weight gain, hyperglycemia, Cushing syndrome and opportunistic infections (7). Additionally, patient non-adherence can compromise treatment efficacy. Vall *et al* (3) reported a case in which poor adherence led to multiple recurrences, ultimately necessitating rescue therapy. While the studies by Alvand *et al* (2) Rajendran *et al* (10) and Oze *et al* (4) reported favorable outcomes with conservative management, the lack of long-term follow-up in these cases raises concerns about the sustained efficacy and reliability of conservative treatment strategies.

A previous meta-analysis of 138 cases undergoing surgery and 358 cases with steroid therapy revealed improved outcomes with surgery (complete response: 90.6 vs. 71.8%; recurrence: 6.8% vs. 20.9%) (13). Combining surgery with steroids further improved results, with a 94.5% complete response rate and a 4.0% recurrence rate (13). A conservative approach was initially employed for the patient in the present study; however, recurrence occurred after 1 year. By contrast, wide local excision of the axillary tail achieved a favorable outcome, with no recurrence observed at the one-year follow-up.

The case described herein enriches the existing body of knowledge by elucidating the presentation, complex diagnosis, treatment options and favorable outcomes of GM in accessory breast tissue, guiding clinicians in recognizing and managing this rare condition effectively. However, it is worth mentioning that a longer period of monitoring would strengthen this report and the conclusions drawn significantly.

In conclusion, GM can occur in accessory breast tissue. While corticosteroids may provide favorable short-term results, they do not necessarily prevent recurrence, whereas surgical management may offer more durable long-term outcomes.

## Figures and Tables

**Table I:** Relevant variables of the reviewed cases.

First author, year of publication	Age, years	Medical history	Use of contraceptives	No. of pregnancies	History of lactation	Duration of lactation (months)	Affected side	Symptoms	Axillary involvement	Treatment approach	Outcome	Follow-up (years)	(Refs.)
Vall, 2025	24	Unexceptional	No	1	Yes	18	Left	Erythema, pain and edema	Yes	Oral corticosteroid followed by rescue therapy due to poor adherence	A few episodes of recurrence with subsequent recovery	N/A	(3)
Salih, 2024	39	Left breast granulomatous mastitis	N/A	3	Yes	37	Right	Right, axillary discomfort, swelling, redness, fever and chills	Yes	Oral amoxicillin and clavulanic acid, followed by excisional biopsy	Recovery	0.5	(1)
Shabani, 2023	38	Unexceptional	No	2	Yes	12	Right	Painful mass, thickened skin and warmth	No	Oral corticosteroid	Recovery	1	(11)
Nakamura, 2022	24	Unexceptional	N/A	Pregnant at the time of presentation	No	N/A	Right	Pain and swelling	Yes	Intravenous piperacillin, drainage and surgical removal	Recovery	N/A	(7)
Oze, 2022	42	Unexceptional	Contraceptive device after her last child	5	N/A	N/A	Right	Swelling, nipple retraction	Yes	Antibiotic therapy and wound dressings	Recovery	0.5	(4)
Alvand, 2022	36	Unexceptional	No	2	Yes	24	Right	Pain, swelling and skin thickening	Yes	Prednisone, naproxen	Recovery	0.75	(2)
Rajendran, 2019	23	Unexceptional	N/A	0	No	N/A	Right	Pain and swelling	Yes	Oral azithromycin and oral prednisone	Recovery	N/A	(10)

N/A, not applicable.

## Data Availability

The data generated in the present study may be requested from the corresponding author.
